# Supplementary material for: Deep learning-enabled detection of rare circulating tumor cell clusters in whole blood using label-free, flow cytometry
Source: Lab Chip. 2024 Mar 8;24(8):2237–52. doi: 10.1039/d3lc00694h (PMC11019838; doi:10.1039/d3lc00694h)
Supplement: LC-024-D3LC00694H-s001 [file LC-024-D3LC00694H-s001.pdf]

Electronic Supplementary Material (ESI) for Lab on a Chip.
This journal is © The Royal Society of Chemistry 2024

Deep Learning-Enabled Detection of Rare Circulating Tumor Cell Clusters in Whole Blood Using Label-free, Flow Cytometry

Nilay Vora[1], Prashant Shekar[2], Taras Hanulia[1,3], Michael Esmail[4,#], Abani Patra[5], Irene Georgakoudi[1,*]

[1] Department of Biomedical Engineering, Tufts University, Medford, MA 02155, USA.

[2] Department of Mathematics, Embry-Riddle Aeronautical University, Daytona Beach, FL, 32114, USA.

[3] Institute of Physics, National Academy of Sciences of Ukraine, Kyiv, Ukraine.

[4] Tufts Comparative Medicine Services, Tufts University, Medford, MA, 02155, USA.

[5] Data Intensive Studies Center, Tufts University, Medford, MA 02155, USA.

[#] Current Affiliation: University of Massachusetts Amherst Animal Care Services, University of Massachusetts Amherst, Amherst, MA 01003, USA.

* Corresponding Author

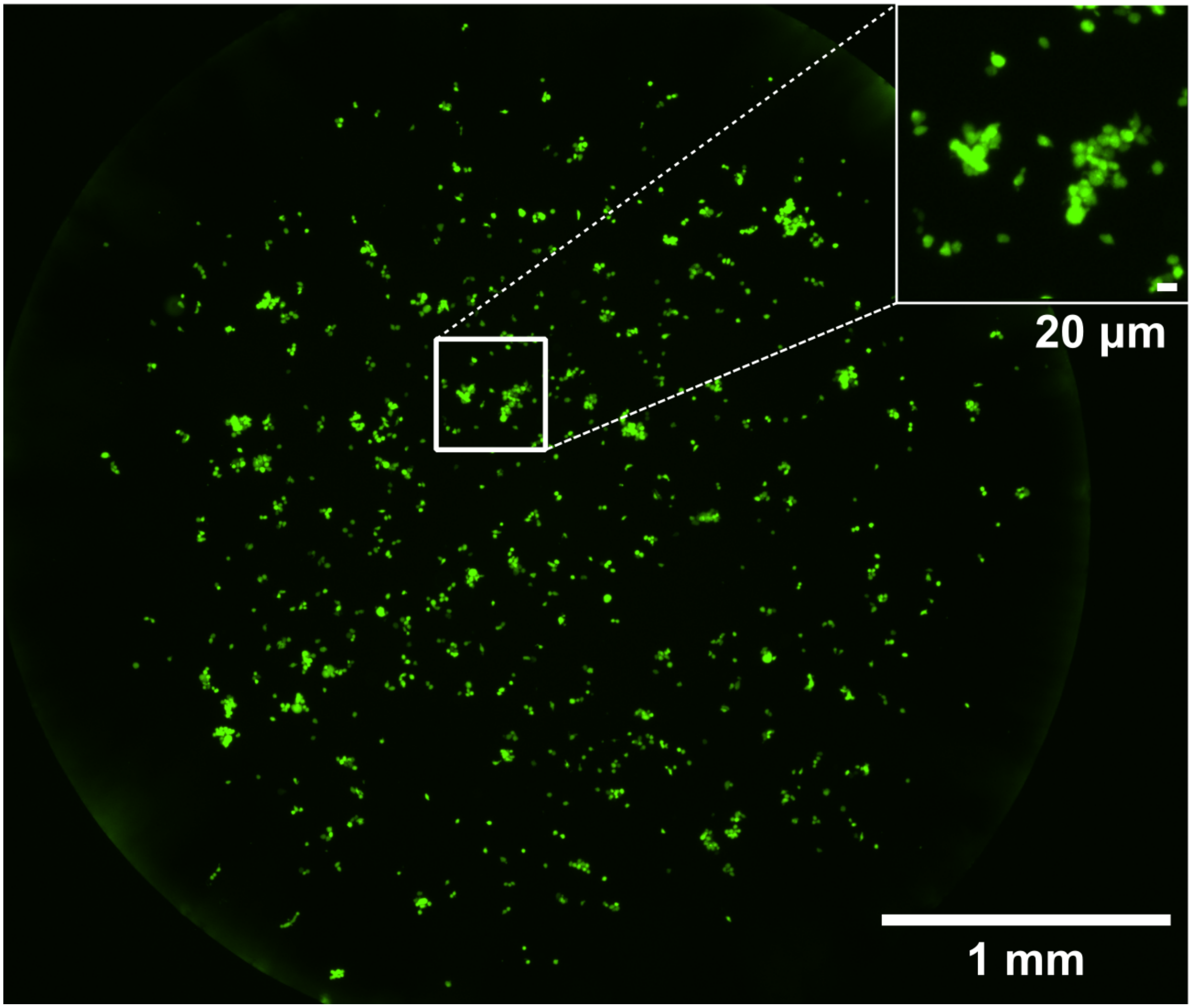

Supplementary Figure S1: Fluorescence image of CTCCs generated prior to spiking in whole blood. CTCs and CTCCs are observed in the spiked samples. CTCCs range from 2-9+ cells in size. Large single cells (~20 µm) are also observed in the image. On average, CTCs are 13 µm in size. The image shown was acquired using a Nikon Eclipse Ti2 with a Plan Fluor 4x PhL DL objective (NA = 0.13).

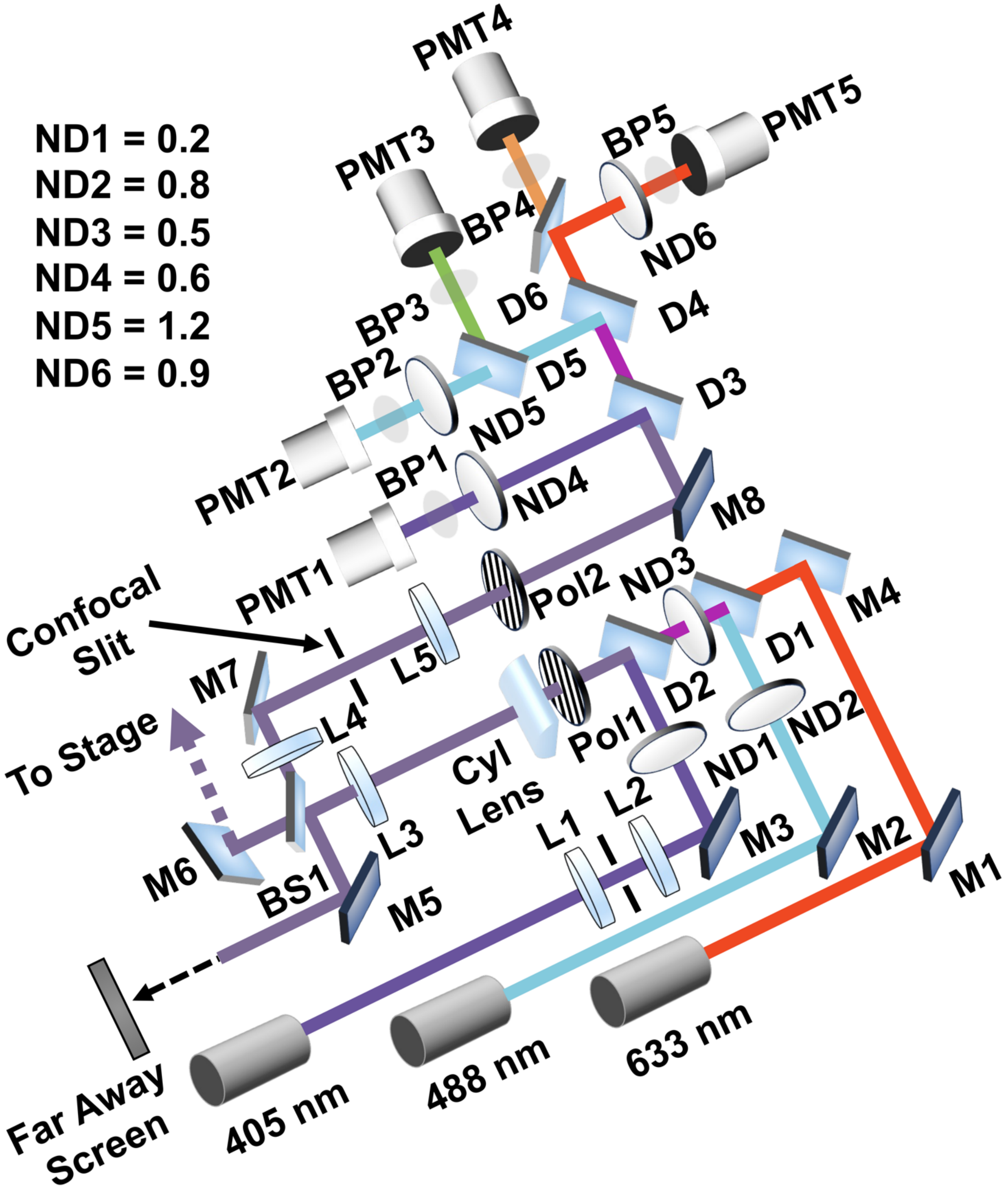

Supplementary Figure S2: Schematic of the optical train for the Back Scatter Flow Cytometer (BSFC). Light is reflected out of page by M6 to the sample stage (see Supplementary Figure S3). *D* dichroic, *L* lens, *M* mirror, *BP* bandpass filter, *BS* beam splitter, *PMT* photomultiplier tube, *Pol* polarizer, *ND* neutral density filter, *Cyl Lens* cylindrical lens.

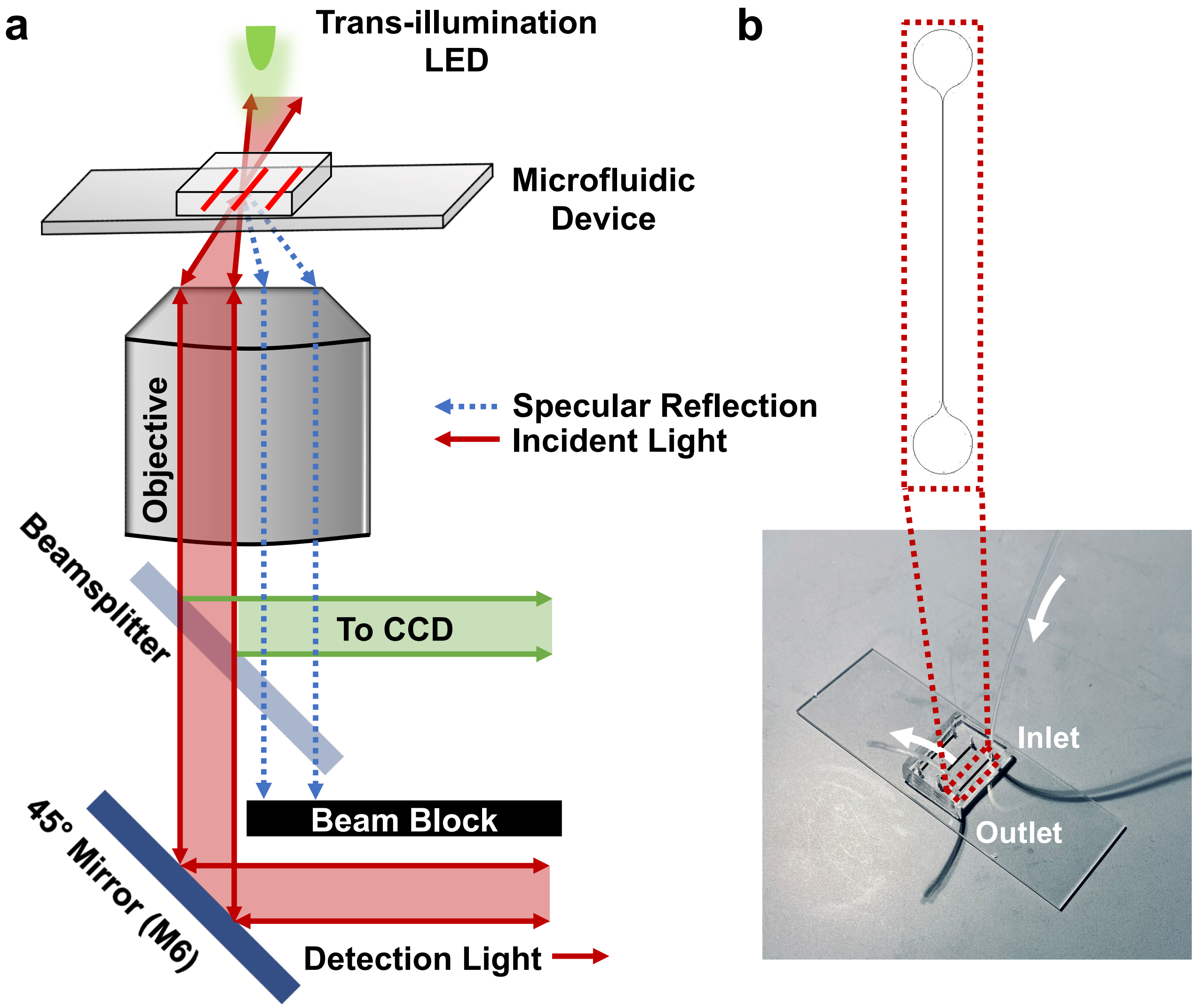

Supplementary Figure S3: **(a)** Schematic of the illumination beam path to the sample stage where the microfluidic device is placed for data collection. Angled illumination is used to improve detection signal-to-noise ratio (SNR) by blocking specular reflection from the glass slide. **(b)** Image of a 30 x 30 μm^2 microfluidic device used for all studies. A zoomed in image of the channel shape is also included (inset).

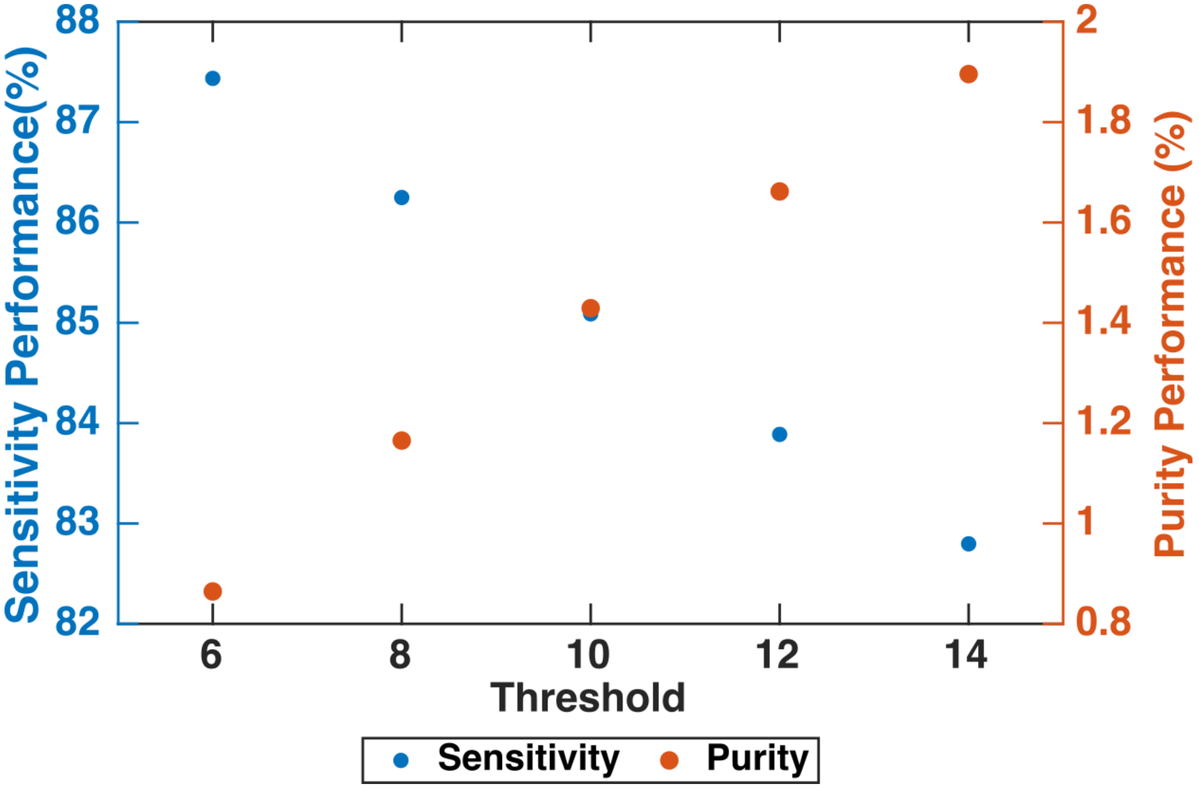

Supplementary Figure S4: Variations in the intensity threshold used by the ROI Detection Algorithm impact the detection sensitivity and purity inversely. A threshold of 10 was selected to maximize both sensitivity and purity.

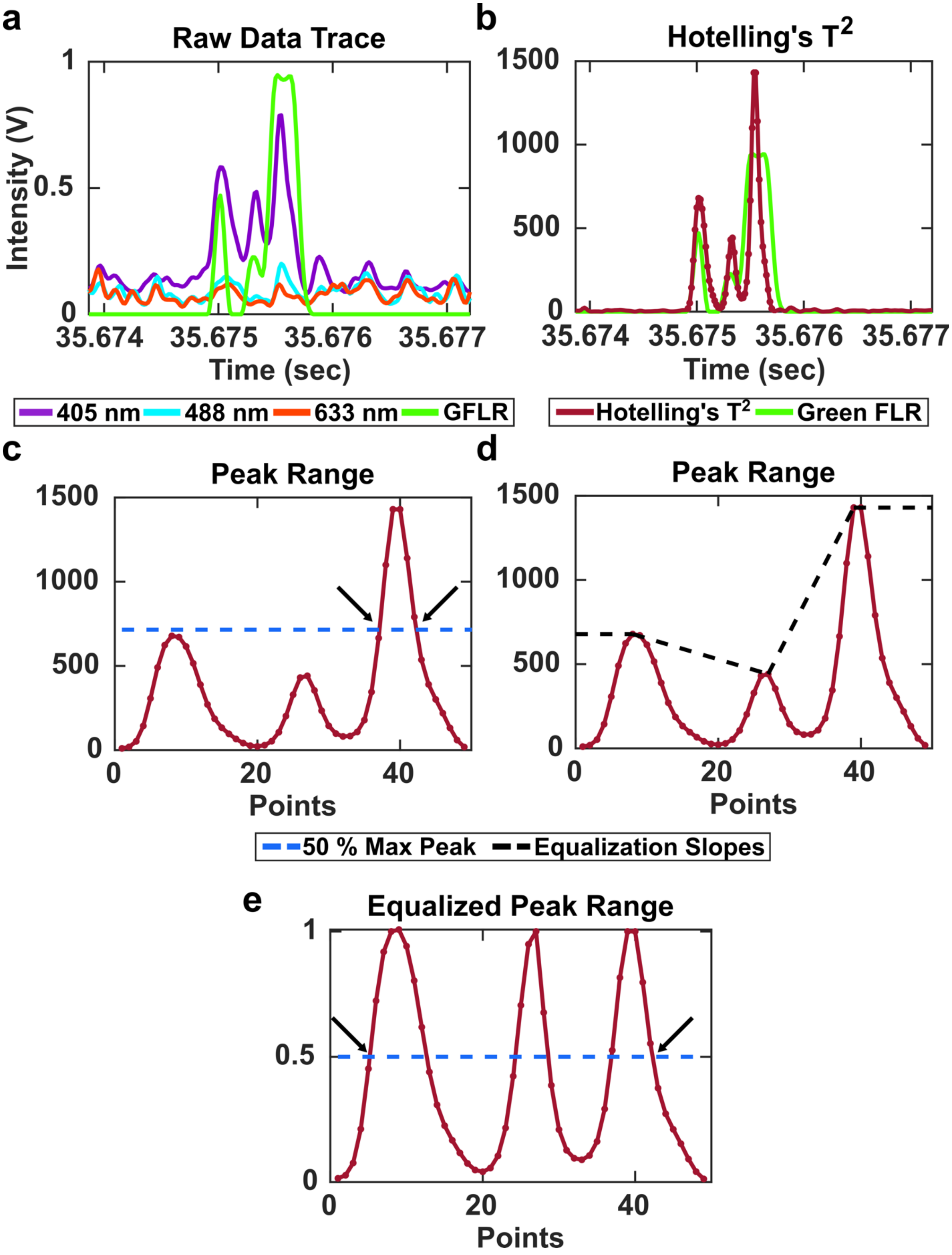

Supplementary Figure S5: **(a)** Raw data trace for a CTCC peak with weak shoulder peaks. **(b)** After PCA and Hotelling's T^2 test, the weak shoulder peaks are still present.

(c) Using our standard intensity threshold algorithm to define the range of points within the cluster, 49 points are selected. The half maximum is calculated (drawn in blue) and full-width-at-half-max (FWHM) is calculated based on the left and rightmost points where the signal crosses the half maximum (indicated with black arrows). This leads to a detected FWHM of 6 points (Corresponding to a narrow single-cell event). **(d)** We use peak equalization to correct for the incorrectly detected narrow peak, calculating the slopes between each peak and normalizing the data based on these slopes. **(e)** The equalized peaks are scaled between zero to one with a half maximum = 0.5 (blue line). The FWHM is again assessed (black arrows) and is now 38 points, correctly indicating a 3-6 cell CTCC event.

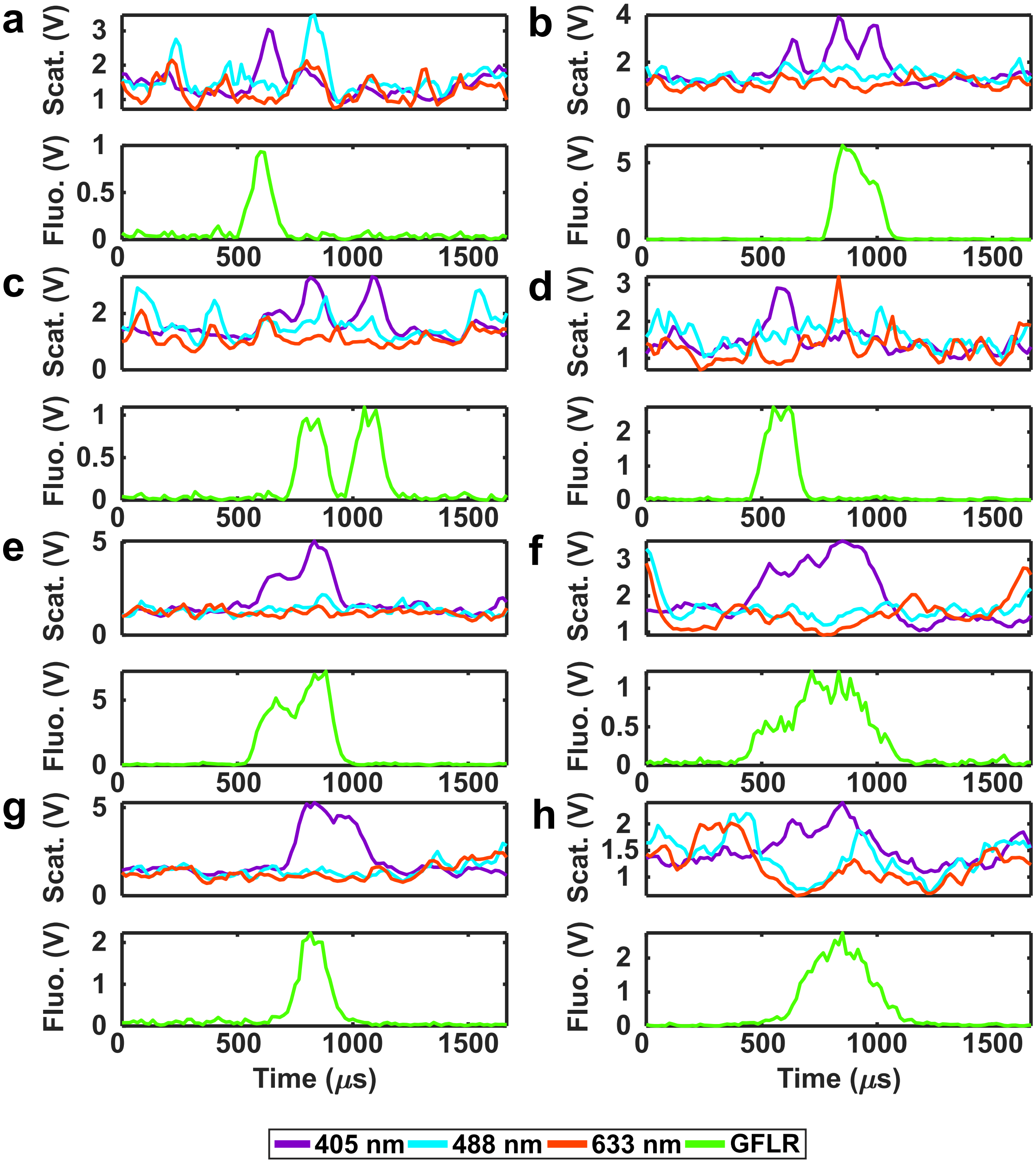

Supplementary Figure S6: Sample raw traces of false negative peaks from the ROI Classification algorithm. False negative events include **(a,d,g)** a 2-cell heterogenous CTC-WBC event, **(b,c)** 3-6 cell heterogenous CTCC, **(e,h)** a homogenous 2-cell CTCC, and **(f)** a 3-6 cell homogenous CTCC. All labels are defined based on FWHM.

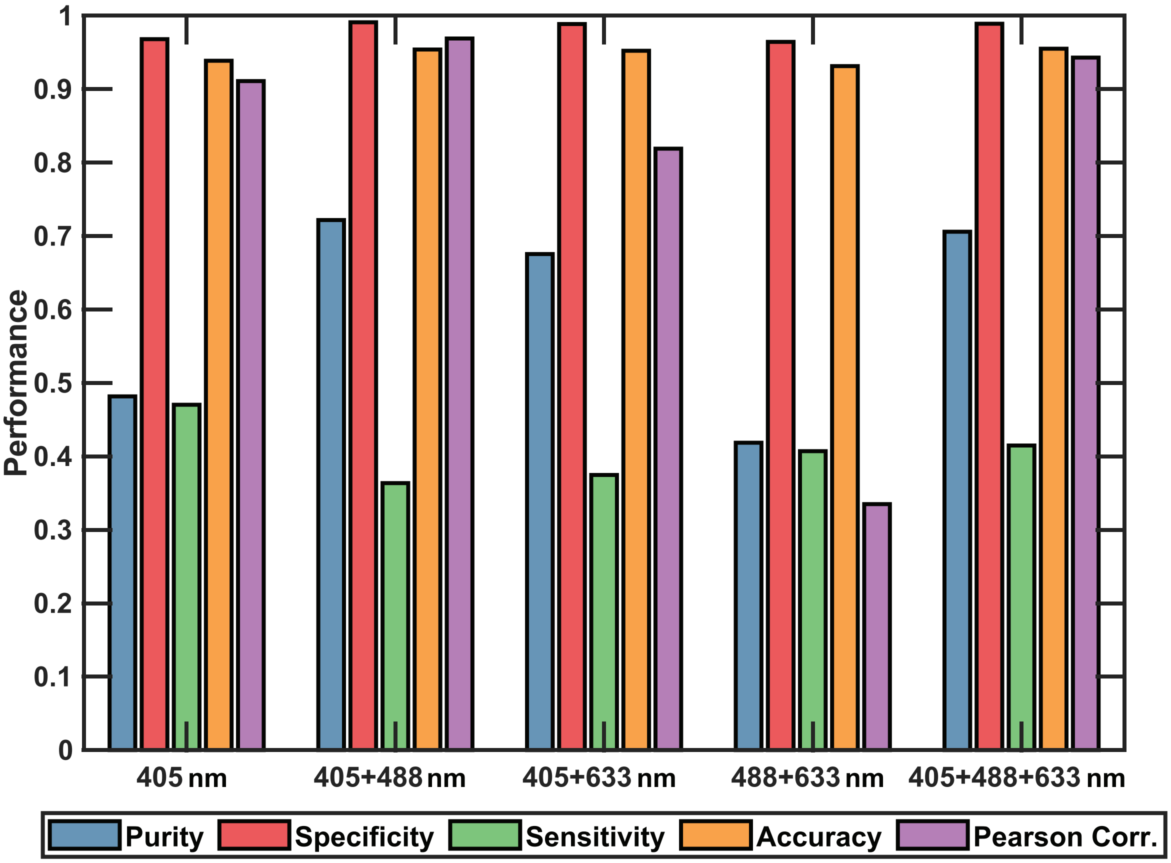

Supplementary Figure S7: Simplified datasets were trained and evaluated using an identical model architecture. Interrogation wavelengths were permuted to identify which laser sources were needed to achieve desirable performance. We observe 405 + 488 nm or 405 + 633 nm can achieve similar performance as 405 + 488 + 633 nm. Further, using 405 nm only could also achieve comparable performance for all metrics outside of detection purity.

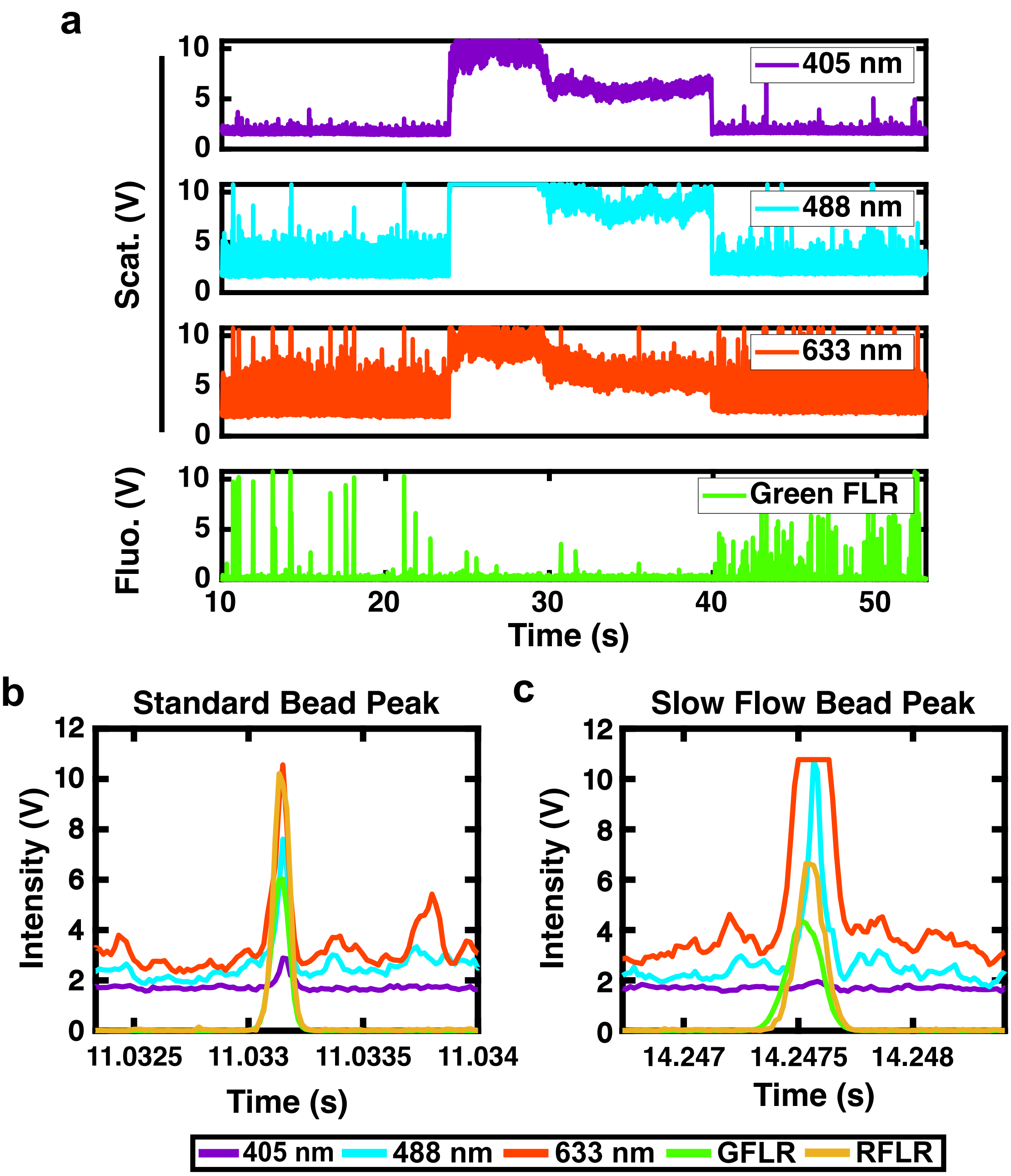

Supplementary Figure S8: **(a)** A representative data trace of the effect of blood clots on signal intensity. From approximately 23 seconds to 40 seconds, due to a blood clot slowing flow within the channel, an air pocket forms resulting in a spike in baseline signal intensity. Such data were removed by replacing the values in the region of the clot with the mean intensity of the signal. **(b)** A representative peak from a fluorescent bead that was spiked into the blood sample prior to flow. Beads could be identified by the red fluorescence signal collected by PMT4 (see Supplementary Figure S2). Fluorescent beads have an average peak width of 7-9 points (as measured in DI Water and in blood) when the flow rate is maintained at 3 □L/min. However, when clots form, slowing flow **(c)**, the peak width of these beads becomes wider (>13 points) due to reduced flow rates.

Supplemental Methods

Alternative Sample Preparation

Data used to train the DeepPeak model was comprised of light scatter data collected from MDA-MB-231 breast cancer cells spiked in whole blood samples drawn from rats. However, additional studies were also carried out on alternative samples to demonstrate the broad utility of the DeepPeak model and BSFC. In the first study, we addressed the differences in blood cell size and composition between different species (rat vs. mouse). In the second study, we examined the utility of BSFC and the DeepPeak model on a different cancer cell line. For both studies, the principal methods outlined in the manuscript were followed.

In the study examining utility of the platform and model on blood samples from different species, fresh whole blood samples were collected from non-experimentally modified mice from other studies via cardiac puncture immediately after CO_2 euthanasia (all relevant animal protocols are listed in the parent text). Blood samples were then spiked in an identical manner as the rat blood studies with MDA-MB-231 breast cancer cells and clusters.

In the study examining performance on different cancer cell lines, CAL 27 epithelial squamous cell carcinoma cells were used for all samples. For this study, GFP-associated CAL 27 cells were cultured on a 10 cm plastic culture plate to 90% confluency in RPMI 1640. To generate CTCCs, an identical procedure was followed for the CAL 27 cells as the MDA-MB-231 cells. GFP-labelled CAL 27 CTCCs were carefully isolated for spiking into whole blood samples. Blood samples were collected from non-experimentally modified rats from other studies as described previously. 100 □L of CTCCs and CTCs of CAL 27 cells were spiked into the blood sample prior to flow through a 30 x 30 □m^2 microfluidic channel.

For both studies, data were collected over the course of five independent days with blood samples from five mice and rats, respectively. All data pre-preprocessing steps remained consistent to those used for studies focused on MDA-MB-231 CTCCs spiked into rat blood. An identical ROI detection algorithm was also implemented for both studies. To assess the performance of the ROI classification algorithm, detected ROIs were either processed through the trained classifier (based on MDA-MB-231 CTCCs spiked in rat blood) or to a retrained classification algorithm (based on the data from the new study). The retrained classification algorithms were generated through transfer learning, using the trained classifier weights as an initializer for a new classifier based on new data.

Transfer Learning

Transfer Learning (TL) is a well-researched method for adapting pretrained models on larger datasets to new smaller datasets[1]. TL is particularly efficient for domain adaptation where data are from related but different domains[1]. Thus, we explored TL as a potential method to highlight that it is possible to optimize in a straightforward manner the

performance of the DeepPeak model on two different datasets acquired from: a) mouse blood spiked with GFP+ MDA-MB-231 CTCCs, and b) rat blood spiked with GFP+ CAL27 cells. To implement TL, the described ROI classification algorithm was recreated before pretrained model weights were loaded. The pretrained weights originated from the algorithm trained of MDA-MB-231 CTCCs spiked into rat blood. Once weights were initialized, the new data was provided to the algorithm and additional training was initiated using identical training parameters as those listed in Methods: ROI Classification Algorithm. Once training was complete, the new model was evaluated on an independent test set to evaluate the performance of TL. As only five days of data was available for both alternative dataset studies, data was split such that two days of data were used for training, one day of data were used for validation, and two days of independent data were used for testing.

Supplemental Results

Validating Performance of the Mouse-DeepPeak Model

Mouse blood cells vary in size and composition compared to rat blood[2]. In mice, there are typically 1 x 10^7 RBCs/□L, 0.5 x 10^6 platelets/□L, and 6 x 10^3 WBCs/□L[2,3]. Compared to rats, this represents an increase in RBCs and platelets and comparable levels of WBCs[4]. Further, mice blood cells are smaller than rat blood cells[3]. Therefore, differences in blood cell sizes and composition between rats and mice were assumed to model similar differences found between rat and human blood. For this study, we collected BSFC data from MDA-MB-231 CTCCs spiked in whole blood samples drawn from mice. Once BSFC data were collected, we first sought to determine the impact of blood cell size and composition on the DeepPeak model. Model weights from the pretrained DeepPeak model (herein referred as Rat-DeepPeak model) were loaded into the ROI classification algorithm and the algorithm was configured to evaluate the new dataset. The direct application of the Rat-DeepPeak model on mouse blood samples led to poor performance as anticipated with a detection sensitivity = 3.2%, detection purity = 15.9%, specificity = 99.9%, and total accuracy = 99.2%. TL was completed as described in the Supplementary Methods: Transfer Learning, and performance of the new model (Mouse-DeepPeak model) was assessed. We observed a significant improvement in both detection sensitivity and purity with sensitivity = 41.4%, purity = 59.7%, specificity = 99.8%, and a total accuracy = 99.4%. These results demonstrate that TL could be utilized to adapt the Rat-DeepPeak model for use on blood samples from other species. The slight decrease in performance between the Mouse- and Rat-DeepPeak model may be a result of the limited dataset available for TL.

Application of DeepPeak Algorithm on CAL27 CTCC

To assess the potential of BSFC to detect different types of CTCCs we performed a preliminary study using the BSFC and DeepPeak model to detect CAL27 CTCCs (CAL 27 is an epithelial squamous cell carcinoma cell line). CAL 27 CTCCs were generated using an identical procedure as listed for the generation of the MDA-MB-231 CTCCs (see Methods: Sample Preparation). BSFC data were collected and processed initially using the pretrained Rat-DeepPeak model architecture to determine if CTCC scattering

signatures between CAL 27 and MDA-MB-231 cells were similar. Using the pretrained model, we observed an improvement in detection purity (89.8%) and detection specificity (99.97%) for CAL 27 CTCCs compared to the MDA-MB-231 CTCCs (69.0% and 98.7%, respectively). However, detection sensitivity significantly decreased for CAL27 CTCCs (4.38%) compared to MDA-MB-231 CTCCs (43.8%). While the improved purity was encouraging, our goal was to maximize both purity and sensitivity. As such, we examined the light scattering properties of CAL 27 and MDA-MB-231 cells to identify if differences in light scattering lead to a loss in sensitivity. To identify the unique scattering properties of light CAL 27 and MDA-MB-231 cells, each cell line was flowed in cell growth media through a clean channel. Detected light scattering signals of the detected cells were then compared to identify if differences in scattering properties existed between the two cell lines. From this study, we observed lower 405 nm light scatter and greater 488 nm and 633 nm light scatter from CAL 27 cells compared to MDA-MB-231 cells (data not shown). It was therefore hypothesized that the difference in the DeepPeak model performance may be attributed to differences in the scattering properties of CAL 27 and MDA-MB-231 cells. To address this difference in scattering properties, we assessed the potential of transfer learning to optimize the model weights to account for differences in the scattering properties of the cells and to improve the performance of the classifier. Here, we applied transfer learning on a small training dataset of three experimental days and evaluated performance on two previously unseen datasets of CAL 27 CTCCs spiked in whole blood. This led to optimization of performance on the classifier with a detection sensitivity of 43.0%, detection purity of 67.6%, specificity of 98.8%, and overall accuracy of 95.7%.

References

1 A. Farahani, B. Pourshojae, K. Rasheed and H. R. Arabnia, *CSCI 2020*, 2020, 344–351.

2 T. Fukuda, E. Asou, K. Nogi and K. Goto, *J. Vet. Med. Sci.*, 2017, **79**, 1707–1711.

3 K. E. O'Connell, A. M. Mikkola, A. M. Stepanek, A. Vernet, C. D. Hall, C. C. Sun, E. Yildirim, J. F. Staropoli, J. T. Lee and D. E. Brown, *Comp. Med.*, 2015, **65**, 96–113.

4 S. L. Delwatta, M. Gunatilake, V. Baumans, M. D. Seneviratne, M. L. B. Dissanayaka, S. S. Batagoda, A. H. Udagedara and P. B. Walpola, *Anim. Model. Exp. Med.*, 2018, **1**, 250–254.
